# Vacation Time, with Hints on Summer Living

**Published:** 1891-09

**Authors:** 


Vacation Time, with Hints on Summer Living. By H. S. Drayton, M. D. New York. Fowler & Wells Co. 1891.

This is the title of a bright little book by H. S. Drayton, M. D., so well known as a writer on popular hygiene, just issued from the press of Fowler & Wells Co , New York.

It is seasonable, filling a niche heretofore vacant, for while we have books giving us good advice about how to live when the weather is cold and the northeast winds blow, this supplies us with a variety of useful information about summer, living, and takes into the account the recreations and diversions that are supposed to belong to warm weather, and into which both old and young enter, according to their circumstances.

The author writes in a pleasant style, and really covers a good deal of ground in a few words. He talks of life at the seaside, in the mountains, of boating and bathing, games, excursions, etc., puts in some very practical hints on eating and dress, and the management of household economies, and has a word of advice to mothers and housekeepers that they cannot but value. Even the stay-at-homes get a thought or two that must be encouraging. As an epitome of summer hygiene the book is so good and practical that they who would read it and follow its suggestions could not but get real profit out of their summering, wherever they might be.

It is sent by mail on receipt of price, 25 cents. Address the publishers, Fowler & Wells Co., 775 Broadway, New York.

